# A Review of Diamond-Blackfan Anemia: Current Evidence on Involved Genes and Treatment Modalities

**DOI:** 10.7759/cureus.10019

**Published:** 2020-08-25

**Authors:** Anshika Tyagi, Apurv Gupta, Anirban Dutta, Pooja Potluri, Badie Batti

**Affiliations:** 1 Medicine, Maulana Azad Medical College, New Delhi, IND; 2 Medicine, Dr. NMB Baruah Nursing Home, Nalbari, IND; 3 Medicine, Jawaharlal Nehru Medical College, Belgaum, IND; 4 Medicine, Alkindi Teaching Hospital, Baghdad, IRQ

**Keywords:** anemia, inherited bone marrow failure, ribosomopathy

## Abstract

Diamond-Blackfan anemia (DBA) is a congenital cause of bone marrow failure predominantly involving the erythroid cell line, with occasional impact on other cell lines. In the vast majority of cases, it is diagnosed by one year of age. We looked at the existing literature on the disease presentation along with established as well as upcoming treatment options. Numerous genes have been identified and extensively studied in the context of their part in the pathogenesis of DBA. Treatment revolves around the use of steroids and regular blood transfusions, with hematopoietic stem cell transplantation reserved for steroid-resistant cases. Newer modalities such as gene therapy, l-leucine, sotatercept, trifluoperazine, SMER28, and danazol are also concisely discussed. The purpose of this article is to review the previous literature on DBA and weigh the role of newer therapeutic agents.

## Introduction and background

Diamond-Blackfan anemia (DBA) is a congenital bone marrow failure syndrome that chiefly affects the erythroid precursors. It presents mostly as severe anemia in infants, along with craniofacial anomalies, poor growth, and various abnormalities of the limb and viscera. The diagnostic investigation is bone marrow smear, which shows a decreased number of erythroid precursors but a normal cell population of myeloid and megakaryocytic lineage [1]. The hematological profile is that of normochromic normocytic or macrocytic anemia [2]. Another evidence of an inherent defect in the pathway of erythropoiesis can be found in the recovery of hemoglobin levels after bone marrow transplantation [3]. Some studies have suggested that not only the erythroid lineage but, in some patients, myeloid and lymphoid precursors are also affected, thus leading to neutropenia and lymphopenia [4].

Since the time DBA was first noticed, we have come a long way in our understanding of this disease. It was first recognized by Josephs in 1936 [5], but a detailed description was provided by Diamond and Blackfan in 1938 [6]. Several studies have been published in the 84 years we have known about DBA, each with a different focus. The objective of this article is to examine the contributions made by these studies to the development of our knowledge of the disease while also highlighting the potential areas of future research concerning the treatment of DBA.

## Review

Epidemiology

DBA registries were set up in North America and the United Kingdom to elucidate the epidemiology, pathophysiology, and prognosis of the condition. The data from the UK registry suggested an annual incidence of five per million live births after a retrospective analysis of 80 cases followed over 20 years (1975-1994) [7]. The prevalence is independent of the gender and ethnicity of the population. The majority of the cases are sporadic except a few that are familial. In a study by Orfali et al., data from 60 families for the evaluation of genetics and familial patterns was collected [8]. Nine out of 60 patients were known to have a family history of DBA. Five of those nine patients with a known family history were elucidated in this study to have autosomal dominant mode of inheritance. Hematological abnormalities were found in family members of 16 out of those 51 patients who were initially thought to be sporadic cases of DBA. Out of 51 cases with no known family history, 35 showed no hematological abnormalities in their family members, confirming their sporadic origin. To summarize, DBA in 58% of the evaluated 60 families was sporadic [8].

Etiology 

Various genetic mutations are responsible for the pathogenesis of DBA (Table 1). The list includes mutations in the genes encoding ribosomal proteins of small and large ribosomal subunits. Common genes and the frequency of their involvement are as follows: RPS19 (25%) [9], RPS24 (approximately 2%) [10], RPS17 [11], RPL5 and RPL11 (6.6% and 4.8%, respectively) [12], and RPS10 and RPS26 (2.6% and 6.4%, respectively) [13].

**Table 1:** Chronology of discovery of ribosomal protein gene mutations in DBA DBA, Diamond-Blackfan anemia

Ribosomal protein gene	Year of discovery
RPS19	1999
RPS24	2006
RPS17	2007
RPL5 and RPL11	2008
RPS10 and RPS26	2010

RPS24 Gene

A genome-wide linkage analysis was performed in a study that had enrolled 215 families with DBA phenotype [10]. A region on chromosome 10 was identified as the cause of the hypoproliferation of erythroid precursors. Further sequencing of this region revealed a nonsense mutation (316C->T) in the gene for ribosomal protein synthesis. This nonsense mutation is conducive to the formation of a truncated ribosomal protein S24 [10].

RPL5 and RPL11

These genes are present on chromosome 1, and their protein products form the large subunits of ribosomes. The involvement of this chromosome in pathogenesis was first described by Heyn et al. in 1974 when they observed pericentric inversion of the first chromosome in a DBA patient with cleft lip and palate [14]. Bearing this in mind, five ribosomal protein genes on chromosome 1 were screened in a study with 196 families, and numerous alterations in the sequences of coding regions of RPL5 and RPL11 were noticed [12]. RPL5 and RPL11 gene mutations were associated with a significant frequency (70% and 67%, respectively) of physical malformations as compared to other gene mutations in DBA (46% in RPS19 mutations) [12].

RPS10 and RPS26

A study conducted in 2010 with the participation of 117 DBA families aimed to accomplish a large extent screen of ribosomal protein mutations in a cohort of DBA patients [13]. Five out of 117 probands had mutations in the RPS10 gene sequence on chromosome 10, and 12 out of 117 probands had mutations in the RPS26 gene sequence on chromosome 12 [13]. RPS10 and RPS26 genes form proteins of small subunits of ribosomes. RPS10 genes were maximally noted to have undergone nonsense mutation and RPS26 predominantly harbored mutations in the translation initiation codon [13].

Pathogenesis

The role of ribosomal protein gene mutations has been studied extensively in the context of RPS19 gene mutation. The RPS19 gene is located on the 19th chromosome. Its protein product is required for the development of 18S rRNA and the integration of pre-40S particles during ribosome biogenesis [15,16]. Flygare et al. demonstrated the effect of reduced expression of RPS19 on ribosomal maturation in the erythropoietic cell line of TF-1 [15], whereas Choesmel et al. demonstrated the same effect on the HeLa cell line [16]. This defect in the synthesis of ribosomes affects the cells, which undergo rapid proliferation and differentiation like erythroblasts [16]. Poor ribosomal biogenesis causes increased nucleolar stress, which further leads to enhanced binding of certain ribosomal proteins to p53 inhibitor MDM2 (murine double minute 2) [17]. This binding results in decreased inhibition of p53 by MDM2. Accumulation of p53 protein causes cell cycle arrest and apoptosis in the erythroid lineage. To explain the exclusive loss of erythroid precursors in DBA, the levels of p53 and p21 protein in erythroid differentiated cells were compared with those in myeloid and megakaryocytic cells. It was noticed that the levels were significantly higher in erythroid cells, thus explaining the lineage specificity of DBA [18]. It is understood now that mutations in ribosomal protein genes besides RPS19 can also bring about deficiency in the biogenesis of ribosomes. For example, RPL26 protein product too is instrumental in the maturation of 18s rRNA and is a regulator of p53 just like RPS19 [19]. Thus, its deficiency has similar effects as RPS19 deficiency.

A study in 2012 had suggested one more mechanism behind erythroid aplasia through RPS19 mutations - an anomaly in the translation of certain mRNA transcripts directly or indirectly responsible for the growth of red blood cells [20]. Rarely, DBA patients may exhibit mutations in GATA1 protein, which is a transcription factor involved in erythroid differentiation of cell lines [21]. This study indicated the role of transcription factors in the pathogenesis of DBA, which should be further explored.

Clinical features

An analysis of DBA’s clinical features was performed by Willig et al. in 1999 using the French registry of DBA patients [22]. A total of 229 patients were followed for 13 years. The diagnostic criterion used in this study was the presence of less than 5% of erythroid precursors in the bone marrow with a background of normal population of other cells. It was observed that 98.7% of cases were diagnosed by the age of five years, with 88% of them being diagnosed by one year of age [22]. Around 40% cases of DBA have physical malformations [7]. Detailed incidences of various physical malformations were described in the study by Willig et al. [22]. The highest recorded incidence was of head malformations (20.5%) followed by eye defects. The least incidence was that of neck deformities [22]. The head malformations included microcephaly, flat nose, cleft palate, microretrognathia, high arch palate, low set ears, anomalies of the ears, low hairline, Pierre-Robin’s syndrome, and others [22].

Complications

The association between DBA and increased susceptibility to cancer had been qualitatively described in many case reports, but the first quantitative analysis of the exact increase in risk was provided by Vlachos et al. [23]. This study described cancer predilection in terms of relative risk and cumulative incidence. The relative risk of DBA patients developing cancer was 5.4 [23]. The gross cumulative incidence of all cancers, except myelodysplastic syndrome, in DBA patients by 46 years of age was 22% [23]. In 2018, Vlachos et al. released another analysis that was enhanced with additional six-year information. The relative risk of cancer as calculated in this report was 4.8 and the cumulative incidence of all the statistically evaluable cancers was 13.7% [24]. The difference may simply be a result of an improved database. The common malignancies in DBA according to the report were colon carcinoma and osteogenic sarcoma [24].

Other complications are related to the treatment of DBA. Long-term use of steroids has its own set of problems such as Cushing's syndrome, steroid-induced cataract, and others. Chronic red cell transfusion lands the patient in iron overload and its consequent troubles such as hemochromatosis. Endocrine complications in DBA have also been studied [25]. The highest percentage was determined to be of adrenal insufficiency and hypogonadism [25]. The frequency of endocrine dysfunction was more in patients who were transfusion-dependent as compared to those on steroids [25].

Diagnosis

The scaffolding on which the current diagnostic criteria are based remains the one described by Diamond et al. in their 1976 review [26]. These classical criteria include macrocytic anemia, presentation before the age of one year, normal counts of leukocytes and platelets, reticulocytopenia, and marrow examination revealing depleted red cell precursors [27]. Supporting criteria include major criteria, which are positive family history and the discovery of a pathogenic mutation, and minor criteria, which are congenital anomalies, raised levels of erythrocyte adenosine deaminase (ADA), which are raised in 85% of cases, increased hemoglobin F, and exclusion of other syndromes of inherited bone marrow failure ( [IBMF] Fanconi’s syndrome, Shwachman-Diamond’s syndrome) [27].

The investigations ordered are mostly to rule out the many different causes of infantile anemia, for example, ABO and Rh incompatibility, drugs and toxins, HIV infection, nutritional deficiencies, and transient erythroblastopenia of childhood. This list is not exhaustive. The presence of specific features such as bone marrow smear picture, associated physical abnormalities, elevated erythrocytic ADA, elevated HbF (fetal hemoglobin), and positive family history guides us toward a diagnosis of IBMFs. The differentiation between the different IBMF syndromes is largely reliant on the presence of genetic mutations specific to each condition.

Treatment

The historical and most utilized treatment in DBA is corticosteroid. The literature suggests that 80% of patients show improvement after the first course of steroids, but the remaining 20% do not (steroid-resistant) and hence are started on red cell transfusion. A follow-up of the patients registered in a DBA registry disclosed that 37% were maintained on steroids and 31% on chronic red cell transfusion [28]. Of the transfusion-dependent patients, 22% were steroid responsive to start with but stopped responding over time and hence had to be started on transfusion [28]. Hematopoietic stem cell transplantation (HSCT) is reserved for patients who have developed transfusion dependence. The advantage of HSCT is that it provides a permanent cure and abrogates the need for regular transfusions. The limitations, however, are that the procedure is associated with graft versus host disease and infections secondary to the use of immunosuppression. The first HSCT for DBA was performed by August et al. in 1976 [29]. A study revealed that HSCT of HLA (human leukocyte antigen)-matched donors yielded better outcomes as compared to alternative donors [30]. The outcomes were also dependent on the pre-transplantation status of iron overload in the patient’s body. The lower the load, the better the survival rate [30]. The development of newer therapies for DBA has been in full swing and warrants a brief discussion.

Gene Therapy

The success of gene therapy was demonstrated in a study in which lymphoblastoid cells were collected from patients with DBA [31]. These cells with RPS19 mutation showed retarded maturation of pre-rRNA, decreased protein formation due to poor ribosomal synthesis, and ribosomal stress manifested as p53 activation. After they were treated with viral vectors carrying the RPS19 gene, these cells recovered their protein synthesis and levels of p53 also dropped [31]. Another study reported the efficacy of gene therapy by exhibiting successful treatment of RPS19-deficient DBA mouse models following gene transfer [32]. Stimulation of RPS19 gene expression in RPS19-deficient cells by the transfer of lentiviral vector with cDNA of elongation factor 1 α short (EFS) promoter has also been documented [33]. This provides further direction to investigation in the field of gene therapy as a therapeutic measure.

L-Leucine

Leucine is an essential amino acid and is a regulator of cellular protein synthesis through the mammalian target of rapamycin (mTOR) pathway [34]. The use of L- leucine in the improvement of erythropoiesis in DBA patients was explored by Pospisilova et al. in 2007 [35]. This principle was further proven by various studies reporting improvement in zebrafish models of DBA and in DBA patients [36,37]. A study of the role of amino acid leucine in the treatment of transfusion-dependent DBA is ongoing under ClinicalTrials.gov (NCT01362595) [38].

Sotatercept (ACE-011)

It is a human dimeric fusion protein consisting of an extracellular domain of activin type IIA receptor and Fc domain of human IgG1 [39]. Activin belongs to the transforming growth factor family. It was first purified from porcine ovarian follicular fluid and was demonstrated to increase the secretion of follicle-stimulating hormone from cultured anterior pituitary cells [40]. The participation of activin A in murine erythropoiesis was confirmed by Shiozaki et al. [41] and it came to be known as an erythroid differentiation factor. Sotatercept has documented efficacy in the improvement of chemotherapy-induced anemia [42]. Bearing in mind all this evidence of the potency of ACE-011, studies were conducted to explore its use in the management of DBA. Zebrafish models of DBA, in which the RPL11 ribosomal protein gene was silenced, showed enhanced erythropoiesis following treatment with RAP-011 (a murine analog of ACE-011) [43]. Therefore, this compound shows promise and is currently being evaluated in a clinical trial for DBA registered under ClinicalTrials.gov (NCT01464164) [44].

Trifluoperazine

It is a calmodulin inhibitor (used as an antipsychotic) that has been proven to be involved in promoting erythroid differentiation in many studies. Calmodulin inhibitors showed efficacy in improving pathogenic phenotypes in zebrafish embryos deficient in RPS29, human cancer cell lines deficient in RPS19, and human cord blood-derived CD34+ cells deficient in ribosomal proteins [45]. The mechanism has been interpreted as follows: trifluoperazine inhibits calmodulin-dependent kinases, which stabilize p53 by phosphorylating it [46].

SMER28

SMER28 is a molecule that is involved in the modulation of autophagy. It was investigated as a therapeutic option in DBA. A drug screen was conducted on induced pluripotent stem cells from DBA patients (reprogrammed fibroblasts from patients with inactivating RPS19 and RPL5 mutations) and SMER28 was identified as a compound that stimulated erythropoiesis [47]. Further studies in this area should be supported.

Danazol 

A case was reported in which use of danazol (synthetic steroid) resulted in the resolution of anemia in a steroid unresponsive DBA patient [48]. No clinical trials aimed at analyzing the therapeutic role of danazol in DBA have been conducted yet and should be considered in the future.

## Conclusions

DBA is an inherited syndrome involving the erythroid cell line. Various gene mutations such as RPS19 and RPS26 have been described in detail in the etiopathogenesis of DBA. The cornerstone of treatment is still corticosteroid, with regular blood transfusions and HSCT reserved for more severe and steroid-resistant cases. Current evidence proves that gene therapy has shown success in mice. L-leucine and sotatercept are presently under trial, and we will soon see some developments. For future research, clinical trials with SMER28 and danazol should be considered. To conclude, with the discovery of detailed pathogenesis of DBA and responsible genes in DBA, new doors to treatment have opened, and we can expect some inclusions in the standard treatment protocol.
